# On Some of the Uses of Hyposulphite of Soda in Medicine

**Published:** 1878-05-20

**Authors:** T. B. Greenley

**Affiliations:** Ky.


### SOUTHERN MEDICAL RECORD.

VOL. VIII. ATLANTA, GA., MAY 20, 1878. NUMBER 5.

EDITORS:

THOMAS S. POWELL, M. D. W. T. GOLDSMITH, M. D. R. C. WORD, M. D.

*R. C. WORD, M. D., Managing Editor.*

SUBSCRIPTION—$2.00 PER ANNUM, IN ADVANCE.

☞ All Communications and Letters on Business connected with THE RECORD, for 1877 and 1878, must be addressed to the Managing Editor.

### *ORIGINAL AND SELECTED.*

### ON SOME OF THE USES OF HYPOSULPHITE OF SODA IN MEDICINE.

BY T. B. GREENLEY, M. D., KY.

I have been using hyposulphite of soda in the practice of medicine for over ten years. The first knowledge I had of its use I think was in 1867, for glanders in horses. The first account that I have noticed of its use was in 1866, by Dr. Leavett, of Germantown, Pa., who treated an obstinate case of intermittent fever with it successfully.

In 1868, Dr. Chubb, of Maryland, and others, used it with more or less success in the treatment of intermittent fever. Prof. Polli, of Milan, seems to have been the first to discover its antiseptic properties, and recommended it in zymotic diseases. He believed it to possess anti-fermentative powers in the blood, and advised its use in septicemia, etc.

From my observation of its medicinal effects, I must express the belief that it possesses the power to arrest, or at least to a very great extent control, septicemia in puerperal fever. I have given it in every case of that disease which has come under my care for the last ten years, and fortunately have not lost a case. Of course I used the other ordinary remedies in that trouble, such as quinia and opium in conjunction with the hyposulphite, but mainly relied on the latter.

I have used it in a few cases of typhoid fever, but more particularly in typho-malarial fever, and can say as I said in regard to puerperal fever, have not lost a case of either; but I do not wish to be understood as relying on this medicine alone in these diseases.

It also possesses anti-periodic properties. I have tested its virtues in this respect in many instances. It may be safely said that it will control the paroxysms of intermittent fever in at least half of the cases that come under our treatment. I think it is better adapted to the treatment of the tertian variety than the quotidian, as you have one well day to administer it in. It should be given in full doses during the interval between the paroxysms, and repeated as often as every three or four hours.

In some cases of remittent fever, when the patients were so much prejudiced against quinia as to refuse to take it, I have relieved them solely with the use of the hyposulphite. In fact, I use it as part of the treatment in all cases of remittent fever, and I think with great benefit. It is doubtless anti-pyrotic as well as anti-periodic in its effects. I have given it successfully in cases of chronic boils, and feel confident it would prove a valuable adjunct in the treatment of psora and some of the allied diseases of the skin.

I have been in the habit of using it as a febrifuge and diaphoretic in most of the inflammatory diseases, especially when the temperature is very high.

It possesses slight laxative properties, and as a general thing is sufficient in that respect in most cases of fever.

Of course in typhoid and typho-malarial fevers, where there is a tendency to diarrhœa, we would exhibit it very cautiously, if at all; but as a general rule, we can give it in small doses without its laxative effect.

It is like all other medicines as regards the quantity to be given at a dose. We must be governed in this respect by the condition of the patient. To an adult, under ordinary circumstances, in a case of high fever, we could give from 10 to 20 grains every 2 to 4 hours with safety.

I usually administer it in solution, say 20 grs. to the ounce of water, and give a large spoonful every two hours. This quantity may be diluted with several times its bulk of water and sweetened, which will very much cover its disagreeable taste. When thus diluted and sweetened, children take it very readily.

I have used the sulphite and bi-sulphite of soda, but greatly prefer the hyposulphite to either. It is equally, if not more effectual, as a remedy in all the diseases in which I have tried them, and far more palatable to take.

It would be a difficult matter to so cover the taste of either the sulphite or bi-sulphite as to induce children to take them.

I omitted to say in the outset that hypo-sulphite of soda had been used in diphtheria. I recollect seeing a communication a few years since from a surgeon U. S. Navy, whose name I have forgotten, published in the *American Journal Medical Sciences.* He spoke very flatteringly of the effects of this medicine in diphtheria, both internally and locally. I have had no opportunity to use it in that disease.

Dr. Constantine Paul, of Paris, in 1867, spoke very highly of hypo-sulphite of soda as a disinfectant. He used it in solution to destroy the bad odor of feces in dysentery; and recommended it as a disenfectant, etc., in fetid lochial discharges. He applies it to the parts by moistening cloths, etc., and says it destroys all offensive odors.

It may be said that this medicine is a hobby with me, but it is not the case. I have been using it too long for a hobby to last. Hobbies usually wear out in less than 10 years.

